# Editorial: Enhancing nutrient profile, safety, and sustainability with fermentation technology

**DOI:** 10.3389/fnut.2025.1571781

**Published:** 2025-02-25

**Authors:** Oluwafemi Ayodeji Adebo, John Gieng, Xi Feng

**Affiliations:** ^1^Centre for Innovative Food Research (CIFR), Department of Biotechnology and Food Technology, Faculty of Science, University of Johannesburg, Johannesburg, South Africa; ^2^Department of Nutrition, Food Science, and Packaging, San Jose State University, San Jose, CA, United States

**Keywords:** fermentation, food safety, sustainability, nutrition, bioavailability, health promoting

## Introduction

The global food system faces unprecedented challenges to meet the nutritional requirements of an ever changing world and growing population, coupled with the need to ensure sustainability and food security. The ancient process of fermentation and subsequent fermented foods have provided vital staples for mankind throughout history. It has evolved over the years with advanced technologies and broader substrate sources. By definition, it is a metabolic driven and transformative process which through microbial activities and enzymatic actions enhances the bioavailability, digestibility, nutritional values, safety and organoleptic properties of foods. Further to this are associated reduction and/or elimination of antinutritive constituents and toxic compounds in food.

This age-long process is experiencing a renaissance through modern scientific understanding and technological advances, offering solutions for enhancing nutrient composition, improving food safety and providing sustainable food sources. This Research Topic on “*Enhancing nutrient profile, safety, and sustainability with fermentation technology*” brings together nine innovative studies that explore different applications of fermentation across a diverse range of food products.

## Nutrient profiles

Fermentation improves the nutritional profile of foods by transforming compounds into bioavailable nutrients and synthesizing bioactive compounds. The study by Zhao et al. used multiple starter culture strains for the fermentation of tomato juice. They reported changes in metabolites such as amino acids, carbohydrates, organic acids, and phospholipids, which led to greater nutritional values and antioxidant capacities. Similarly, the study by He et al. used *Pleurotus ostreatus* mycelium in solid state fermentation of soybeans and reported an increase in protein, lipid, and phenolic content and a reduction in dietary fiber and starch. The study by Maleke et al. showed that fermentation improved the acidity, fiber content, and antioxidant properties of sorghum and mopane worm flour, while also improving their water and oil holding capacities and dispersibility. These findings are consistent with other research reporting that fermentation improves functional properties in underutilized grains (1).

Reducing anti-nutritional factors is another key benefit of fermentation. The study by Nsabimana et al. combined fermentation with soaking and germination of maize and reported a significant reduction in phytate content. As a result, there was a significant improvement in iron and zinc bioavailability, reflecting the greater potential of fermentation to improve mineral bioavailability while reducing anti-nutritional factors (1). Similarly, Jia et al. developed a novel low-salt fermentation kohlrabi method, which improves its palatability and nutritional profile while minimizing risks of sodium intake.

Innovative techniques further highlight the potential of fermentation. The study by Kurchenko et al. used oligochitosans, low-molecular weight derivatives of chitosan, to stimulate lactic acid production and improve fatty acid profiles in fermented milk. This technique improves nutritional properties, which is consistent with the findings of other fermentation studies (2). The study by Murakami et al. used a novel double saccharification process to enrich amazake with isomaltose, a functional carbohydrate related to gut health. This supports the ability of fermentation to increase the availability of bioactive nutrients (1). The study by Amanipour et al. used fermentation along with dehulling and germination in gray pea flour. They reported an increase in protein and polyphenol content and antioxidant capacity. These findings are consistent with fermentation improving the antioxidant capacity and availability of bioactive compounds (3).

## Safety

Fermentation contributes to food safety through the production of certain metabolites and bio-preservative compounds. The study of Kurchenko et al. investigated the influence of chitosan on the growth and productivity of *L. bulgaricus* in the presence of chitosan and its derivatives. They reported the beneficial role of chitosan, accelerating the synthesis of lactic acid and an increase in shelf life of milk. Findings from Murakami et al. showed the beneficial effects of saccharified amazake in the gut. Such improved gut health can prevent colonization of pathogenic bacteria and by extension enhanced resistance to foodborne pathogens. Likewise, the reported increase in antioxidant capacities and the presence of certain non-volatile metabolites in fermented soybean (using oyster mushroom) (He et al.) has potential food safety implications. Such antioxidant related compounds can help mitigate the growth of pathogenic microorganisms and prevent spoilage of food through inhibition of oxidation processes. These subsequently contribute to the overall safety of food.

While not explicitly mentioned in some of the studies, competitive exclusion of pathogenic microorganisms occurs during fermentation. Proliferation of fermenting microorganisms also leads to the reduction, degradation and sometimes total elimination of toxic components. Such a reduction in anti-nutrients was demonstrated in the study of Maleke et al. which might have extended to potential toxic constituents in the substrates used.

## Sustainability

Fermentation is a potential solution to reduce food waste and increase sustainability. In this Research Topic, He et al. investigated antioxidant capacities and non-volatile metabolites changes after solid-state fermentation of soybean using oyster mushroom (*Pleurotus ostreatus*) mycelium. This study explored the usage of *P. ostreatus* mycelium to enhance the quality of soybean products and discussed the potential strategies to improve soybean properties for creating meat analogs. Nsabimana et al. studied the enhancement of iron and zinc bioavailability in maize by fermentation with or without soaking and germination, which can reduce micronutrient deficiencies in developing regions.

Some agricultural products are overlooked, such as gray peas and insect protein. Amanipour et al. investigated the dehulling, germination, and fermentation of the bioactive and functional properties of gray pea flour. The results could encourage industries and farmers to increase gray pea production and processing. Maleke et al. studied the effect of fermentation, malting, and ultrasonication on sorghum, mopane worm, and *Moringa oleifera*. The results indicated that ultrasonication was more effective in improving the nutritional value of the samples, followed by fermentation. The blend of bioprocessed flours with various nutritional and health properties can help address the challenges of malnutrition.

Meanwhile, innovative fermented technology can also improve human health, such as decreasing salt intake. Jia et al. investigated the non-volatile metabolites in a low-temperature and low-salt fermented Chinese kohlrabi (LSCK). Future research in optimizing the LSCK processing could decrease the salt contents in this traditional Chinese food as well as decrease water usage in traditional Chinese Hohlrabi manufacturing as desalting uses a high amount of water.

## Conclusions

The studies in this Research Topic have highlighted the versatility of fermentation in enhancing nutrient profile, safety and other possibilities for better and sustainable food systems. The diverse studies presented also open new pathways for innovation in food production systems, ultimately working toward a more sustainable and nutritious food supply for future generations. As we confront global challenges of food security, malnutrition, and environmental sustainability, there is still a need for more studies to exploring novel microbial strains, investigating the scalability of these findings and others, as well as developing sustainable integration strategies to meet the needs of an ever-growing population.
